# The association of vasoactive-inotropic score and surgical patients’ outcomes: a systematic review and meta-analysis

**DOI:** 10.1186/s13643-023-02403-1

**Published:** 2024-01-06

**Authors:** Yan-ting Sun, Wei Wu, Yun-tai Yao

**Affiliations:** 1 Department of Anesthesiology, Baoji High-Tech Hospital, Shaanxi, 721000 China; 2 Department of Anesthesiology, Fuwai Hospital, National Center for Cardiovascular Diseases, Peking Union Medical College and Chinese Academy of Medical Sciences, Beijing, 100037 China

**Keywords:** Vasoactive-inotropic score, Clinical outcome, Surgery

## Abstract

**Background:**

The objective of this study is to conduct a systematic review and meta-analysis examining the relationship between the vasoactive-inotropic score (VIS) and patient outcomes in surgical settings.

**Methods:**

Two independent reviewers searched PubMed, Web of Science, EMBASE, Scopus, Cochrane Library, Google Scholar, and CNKI databases from November 2010, when the VIS was first published, to December 2022. Additional studies were identified through hand-searching the reference lists of included studies. Eligible studies were those published in English that evaluated the association between the VIS and short- or long-term patient outcomes in both pediatric and adult surgical patients. Meta-analysis was performed using RevMan Manager version 5.3, and quality assessment followed the Joanna Briggs Institute (JBI) Critical Appraisal Checklists.

**Results:**

A total of 58 studies comprising 29,920 patients were included in the systematic review, 34 of which were eligible for meta-analysis. Early postoperative VIS was found to be associated with prolonged mechanical ventilation (OR 5.20, 95% CI 3.78–7.16), mortality (OR 1.08, 95% CI 1.05–1.12), acute kidney injury (AKI) (OR 1.26, 95% CI 1.13–1.41), poor outcomes (OR 1.02, 95% CI 1.01–1.04), and length of stay (LOS) in the ICU (OR 3.50, 95% CI 2.25–5.44). The optimal cutoff value for the VIS as an outcome predictor varied between studies, ranging from 10 to 30.

**Conclusion:**

Elevated early postoperative VIS is associated with various adverse outcomes, including acute kidney injury (AKI), mechanical ventilation duration, mortality, poor outcomes, and length of stay (LOS) in the ICU. Monitoring the VIS upon return to the Intensive Care Unit (ICU) could assist medical teams in risk stratification, targeted interventions, and parent counseling.

**Systematic review registration:**

PROSPERO CRD42022359100.

**Supplementary Information:**

The online version contains supplementary material available at 10.1186/s13643-023-02403-1.

## Background

Timely initiation of vasoactive and inotropic medications is crucial for hemodynamic management in both surgical settings and intensive care unit (ICU) [1–3]. While effective in improving hemodynamic parameters, these medications also have significant side effects, including increased myocardial oxygen consumption, myocardial ischemia, arrhythmia, ischemic perfusion injury, and multi-organ failure [4, 5]. Emerging evidence suggests that excessive doses of these drugs could be detrimental during surgical procedures [6, 7]. The severity of illness quantification is vital for critical care physicians, as it can guide patient prognostication and family counseling.

The vasoactive-inotropic score (VIS), first proposed in 2010 [1], has since been extensively used to quantify cardiovascular support after cardiac surgery in pediatric patients. Davidson et al. [8], in a study on 70 infants (≤ 90 days of age), found that a higher VIS at 48h post-cardiothoracic surgery was strongly correlated with prolonged mechanical ventilation and extended stays in both the ICU and the hospital. This easy-to-calculate bedside tool has been validated as an independent predictor of adverse outcomes, such as duration of mechanical ventilation, length of stay (LOS) in the ICU and hospital, and mortality [1, 8, 9]. For cardiac surgical patients, the VIS represented a significant advancement in assessing hemodynamic needs at specific time points post-surgery, facilitating meaningful comparisons across patients and institutions. Not limited to pediatric settings, the VIS also predicts adverse postoperative outcomes in adults. For instance, a study by Koponen et al. [10] conducted a noteworthy study that aimed to retrospectively evaluate the association between the highest VIS in the first 24 h post-ICU admission and a composite poor outcome in 3213 adult cardiac surgical patients, elucidating a linear increase in the odds of adverse primary postoperative outcomes with escalating ICU-VIS scores.

To date, most studies regarding the application of the VIS have been conducted in the respective authors’ countries and regions [11–14]. A recent systematic review published by Belletti et al. [15], which searched from 2010 to 2019, examined the evolution, clinical utility, and pitfalls of the VIS. Despite differing VIS calculation timings and cutoff values across studies, all concurred on its value as an outcome predictor. However, no comprehensive qualitative or quantitative reviews have yet been conducted specifically on the association between VIS and surgical patient outcomes. This study aims to address this gap, with primary outcomes focusing on the quantitative relationship between the VIS and surgical patient outcomes. Secondary outcomes will consider the optimal the VIS cutoff value for predicting these outcomes.

##  Methods

###  Protocol and registration

The systematic review and meta-analysis followed the Preferred Reporting Items for Systematic Reviews and Meta-Analysis (PRISMA) guidelines [16] and were registered at PROSPERO (registration number CRD42022359100) [17].

###  Search strategy

Three reviewers (YTS, WW, YTY) independently searched databases including PubMed, Web of Science, EMBASE, Scopus and Cochrane Library, Google Scholar, and CNKI from November 2010, when the VIS was first published, to December 2022. To maximize the chances of identifying relevant studies, different combinations of search words were used as follows: “(vasoactive and inotropic score) AND (mortality OR morbidities OR complications).” No restrictions were used.

###  Inclusion and exclusion criteria

We included the following studies: (1) population, pediatrics and adults undergoing any type of surgery; (2) study type, any; (3) outcomes, studies evaluating the association between the VIS and short- or long-term patient outcomes; (4) publication type, any article type; and (5) language, published in English.

Excluded studies were as follows: (1) review articles, case reports, guidelines, conference abstracts, letters, or studies with insufficient data; (2) duplications; (3) studies with incomplete or incorrect data and those not analyzing the association between VIS and outcomes; and (4) grey literature.

###  Study selection

After deleting duplicates records by using EndNote X7 reference management software, two of the authors (WW and YTS) independently examined the titles and abstracts of all potentially relevant studies and retrieved the full-text records for eligibility. Two reviewers (YTS and WW) independently reviewed the titles and abstracts of all identified studies for eligibility, followed by a full text review. Disagreement on inclusion was resolved by consensus and after discussion with the senior reviewer (YTY).

###  Data extraction

The data was independently extracted by YTS and WW into an Excel table, including study information (first author, publication year, country, years of collection, sample size, type of surgery), patient demographics (age, gender), the VIS data (time points of collection, cut-off value, validity of the VIS), and cross-sectional association between the VIS and outcomes (adjusted or unadjusted odds ratio (OR) with corresponding 95% confidence intervals (CI)). The OR value adjusted to the maximum extent for potentially confounding variables was selected, for only one model could be selected from studies reporting more than one adjusted mode. Predictive validity was defined as the ability of the VIS to predict patient outcomes, including the receiver operator characteristic (ROC) and area under the curve (AUC), sensitivity, and specificity. Disagreements were resolved by discussion with author (YTY).

###  Study quality assessment

Quality assessment was conducted independently by two reviewers (YTS and WW) using the Joanna Briggs Institute (JBI) Critical Appraisal Checklists for analytical observational studies [18]. Any disagreement in opinion regarding quality was resolved by discussion consensus with a third investigator (YTY). JBI Critical Appraisal Checklist contains 11 questions for cohort studies, 8 for cross-sectional studies and 10 for case control studies. All questions to determine the potential risk of bias can be answered with yes, no, unclear, or not applicable. If the answer is yes, the question is assigned a score of 1. If the answer is no, unclear, or not applicable, it is assigned a score of 0. A score of 4 to 6 indicates moderate quality, whereas as score of 7 or more indicates high quality.

###  Statistical analysis

The primary outcomes focused on the quantitative review of the association between the VIS and patient outcomes. Meta-analysis was conducted if two or more studies provided the same effect concerning the VIS. Adjusted ORs from multivariate aggressive analysis, along with their respective 95% of CIs, were extracted from each of the studies. The reported ORs were converted into log(OR), and the 95% CIs were transformed into standard errors (SE) using a random-effects model to pool the data [19, 20]. A random-effects meta-analysis was used because of the expected heterogeneity. Heterogeneity was assessed using the Q statistic and an* I*^2^ index score, with *P* < 0.10 and *I*^2^ > 50% considered statistically significant. Publication bias was evaluated through a visual inspection of funnel plots. Sensitivity analysis was considered to examine the influence of each study on the stability of the meta-analysis results. Subgroup analysis was attempted if possible to address the potential sources of heterogeneity. All analysis were performed using Review Manager 5.3 (Cochrane Collaboration, Denmark).

The secondary outcomes focused on the qualitative review of the predictive value of the VIS. In this review and meta-analysis, apart from one non-cardiac surgery [21], all other included studies were cardiac surgery including the large number of different surgical procedures, and almost all of the studies included were retrospective and the outcome variable varied from each study. We could not pool sensitivity and specificity estimates across different VIS cut-off values, as these varied between studies. Therefore, we summarized the results of each article individually (Table 2).

##  Results

###  Search results

**Fig. 1:**
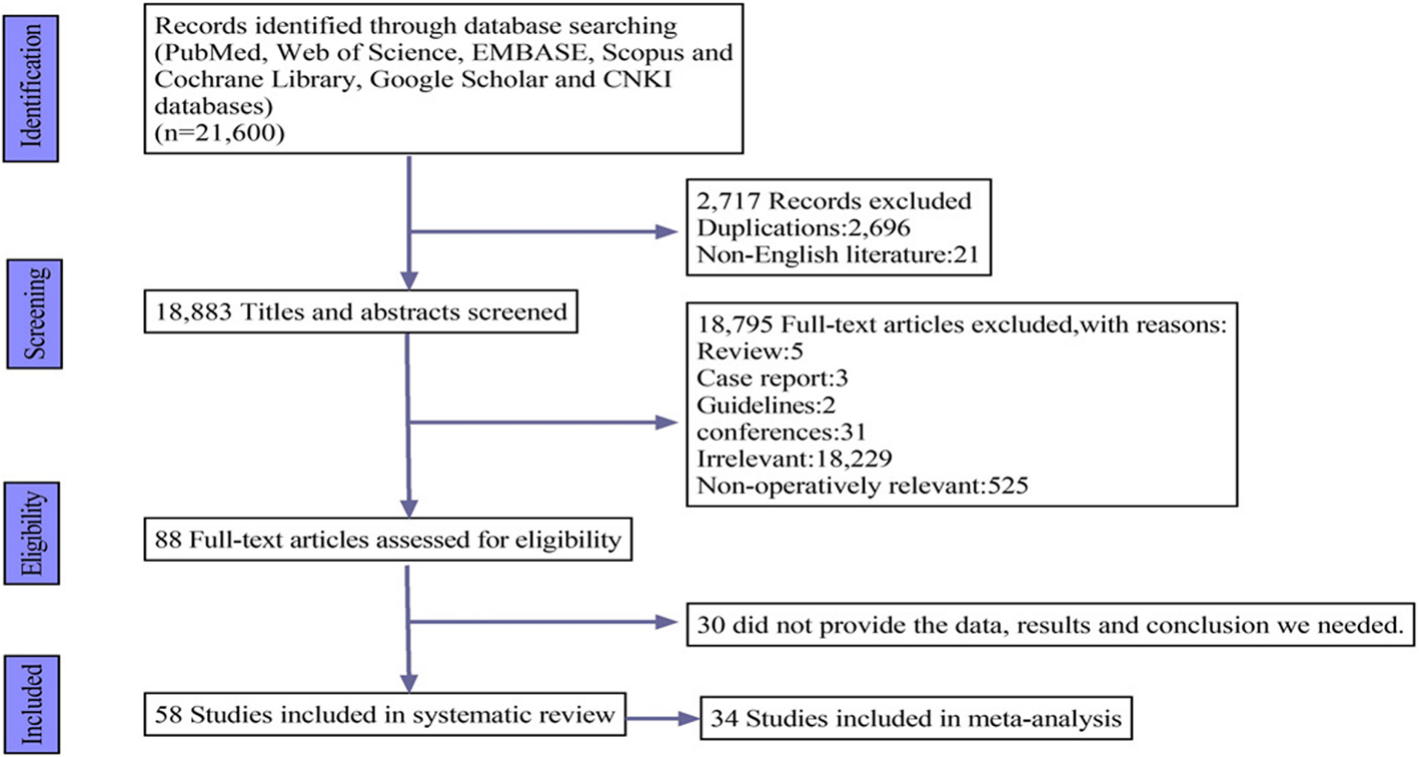
PRISMA flow diagram of study selection

Figure 1 illustrates the different phases of the search and selection processes. A total of 21,600 records were identified, of which 58 studies met the inclusion criteria and 34 studies were included in the meta-analysis. Apart from one prospective randomized study [22] and one retrospective secondary analysis of an RCT [23], all other included studies were observational and had low to moderate risk of bias according to the assessment results of JBI checklist scores (Table S1–S3).

###  Study characteristics

The VIS was applied in studies across 14 countries, with the majority conducted in the USA (*n* = 22), followed by China (*n* = 9), India (*n* = 5), Turkey (*n* = 5), Germany (*n* = 4), Korea (*n* = 3), Russia (*n* = 2), Spain (*n* = 2), Canada (*n* = 1), Finland (*n* = 1), France (*n* = 1), Japan (*n* = 1), Canada (*n* = 1) and Australia (*n* = 1). Data from a total of 29.920 patients with the VIS were included, of which 11.409 (38.13%) were female patients, with a mean age range of 6 (1–90) days to 68 (19–90) years. The number of participants ranged from 32 to 8543 in each included study. The VIS was recorded in the early postoperative period, including 24 h, 48 h, and 72 h. Among the fifty-eight studies, 42 involved pediatric populations, and 16 involved adult patients (Table 1). As shown in Table 2, the optimal cutoff value of the VIS as a predictor of outcomes varied widely, and none of the studies reported an identical VIS cutoff value. The ROC area ranged from 76% to 94%, with sensitivity from 53% to 90% and specificity from 74% to 88%.

**Table 1:** Characteristics of included studies

Study	Country	Design	Time period	Surgery	*N*	Mean age	Female, %	Time point of the VIS
**Pediatrics**								
Gaies et al., 2010 [1]	USA	Retrospective Single-central	2007–2008	CHD surgery with CPB	173	0 to 6 mon	45	VIS 24 h, VIS 48 h (mean, max) after cardiac surgery
Dilli et al., 2019 [3]	Turkey	Prospective cohort Single-central	2016–2019	Cardiac surgery	119	15 (9–31) days	46.70	VIS 72 after cardiac surgery
Pérez-Navero et al., 2019 [2]	Spain	Prospective observational study	N/A	CHD surgery	117	39.8 ± 52.4 mon	N/A	VIS2, 12, 24, and 48 h after cardiac surgery
Davidson et al., 2012 [8]	USA	Prospective Single-central	2009–2010	Cardiovascular surgery	70	N/A	39	VIS 24, VIS 48 (max), VIS 72 h after surgery
Scherer et al., 2016 [24]	USA	Prospective Single-central	2015–2015	CHD surgery	164	9.03 (2.6–58) mon	38	VIS 6 h, VIS 12 h, VIS 24 h, VIS 48 h after cardiac surgery
Tadros et al., 2020 [25]	USA	Retrospective Single-central	2011–2018	Heart transplantation	104	7.2 (0.5–15.4) yr	38.5	VIS 24 h,VIS 48 h (mean, max) after orthotopic heart transplantation
Garcia et al., 2016 [9]	USA	Retrospective Single-center	2008–2014	CHD surgery	149	14.8 ± 2.4 yr	34.9	VIS 48 h (max) after surgery
Kim et al., 2015 [26]	Korea	Retrospective Single-central	2005–2012	ASO for TGA	115	6 (1–90) days	30.4	VIS before operation
Parmar et al., 2017 [27]	India	Prospective Single-central	2015–2016	Ventricular septal defect closure	135	26.7 mon	45.2	VIS during intraoperative
Butts et al., 2012 [23]	USA	Retrospective secondary analysis of RCT Single-central	2007–2009	Cardiac surgery	76	7 days	NA	VIS 36 h (max) after surgery
Bangalore et al., 2017 [28]	USA	Retrospective Single-central	2010–2011	Cardiac surgery with CPB	167	2.9 ± 6.0 yr	47	VISmean 24 h, VIS 24 h (max), VIS 48 h (max), VISmean 48 h after surgery
Little et al., 2014 [29]	USA	Retrospective Single-central	2001–2010	Norwood operation	64	6.5 (5–8) days	35.3	VIS 48 h (max) after surgery
Crow et al., 2014 [30]	USA	Retrospective Single-central	2002–2011	CHD surgery	255	122 (0–362) days	41.6	VIS0–72 h (max) after surgery
Raatz et al., 2019 [31]	Germany	Retrospective Single-central	2014–2016	CHD surgery	745	7 (2–55) mon	42.3	VIS 0, VIS 48 ,VIS 72 h after surgery
Kuraim et al., 2018 [32]	Canada	Prospective Single-central	2003–2012	Cardiac surgery	565	38.7 weeks	38	VIS pre-operative, VIS 24 h (max) post-operative
Beken et al., 2021 [33]	Turkey	Retrospective Single-central	2015–2018	Cardiac surgery	199	2 (1–7) days	31.7	VIS 24 h (max) after surgery
Luo et al., 2020 [34]	China	Retrospective Single-central	2010–2018	TCPC surgery with CPB	504	2.3–36 yr	NA	VIS (max) during surgery
Zhang et al., 2018 [35]	China	Retrospective Single-central	2010–2017	ALCAPA surgery	71	12 (8.8–48) mon	40.8	VIS (max) during surgery
Schroeder et al., 2018 [21]	Germany	Retrospective Single-central	2013–2015	Congenital diaphragmatic hernia	34	7.15 days	23.5	VIS 24 h (mean) before extubation
Kulyabin et al., 2020 [22]	Russia	Prospective randomized study Single-central	2016–2019	Aortic arch reconstruction	45	3–30 days	NA	VIS 24 h, VIS 48 h, VIS 36 h after surgery
Chen et al., 2020 [36]	China	Retrospective Single-central	2012–2016	VA-ECMO-treated CABG for PCS	121	624 (55–67) mon	21	VIS 6 h before ECMO cannulation
Miletic et al., 2015 [37]	USA	Retrospective Single-central	2009–2013	Cardiac surgery with CPB	222	107.7 (3–358) days	47	VIS 0 h, VIS 48 h after surgery
Murin et al., 2020 [38]	Germany	Retrospective Single-central	2014–2018	CHD surgery with CPB	615	NA	NA	VIS 24 h after surgery
Ödek et al., 2016 [39]	Turkey	Prospective Single-central	2012–2014	Cardiac surgery for CHD	99	13 (3–192) mon	48	VIS at PICU admission
Sanil et al., 2013 [40]	USA	Retrospective Single-center	2004–2010	Heart transplant surgery	51	1.3 yr	51	max VIS 24 h, VIS 48 h after OHT
Gaies et al., 2014 [41]	USA	Prospective, multi-center	2011–2012	Cardiac surgery with CPB	391	84 (9–165) days	45	VIS 48 h after surgery
Kumar et al., 2014 [42]	India	Retrospective Single-center	2012–2013	Cardiac surgery for CHD	208	66.94 mon	27.9	max VIS 48 h after surgery
Kulyabin et al., 2020 [43]	Russia	Retrospective Single-center	2008–2018	Aortic arch reconstruction with CPB	121	29 (3–270) days	42.5	VIS 24 h after surgery
Alam et al., 2018 [44]	India	Prospective Single-central	2015–2015	Surgery for CHD with or without CPB	574	< 1 yr	41.1	VIS after surgery
Tabbutt et al., 2019 [45]	USA	Retrospective Multi-center	2014–2016	Cardiac surgery	8543	NA	44.9	max VIS 2 h after surgery
SooHoo et al., 2018 [46]	USA	Retrospective Single-center	2009–2015	Norwood operation (stage I palliation)	95	4.5 (3–6.5) days	32.6	VIS 0 h, VIS2 4 h, VIS 48 h, max VIS 7 h after surgery
Campbell et al., 2020 [47]	USA	Prospective Single-central	2012–2016	Cardiac surgery with CPB	34	2.5 (0.6–12.0) yr	47	VIS 2 h, VIS 2–24 h, VIS 24–48 h post-CPB
Talwar et al., 2018 [48]	India	Retrospective Single-center	2003–2013	Bidirectional Glenn procedure	215	5.29 ± 5 yr	29	VIS 24–72 h after surgery
Ödek et al., 2018 [49]	Turkey	Retrospective Single-center	2008–2013	Cardiac surgery	126	10 (1.5–168) mon	50.8	VIS at PICU admission
Sun et al., 2022 [50]	China	Retrospective Single-center	2021–2021	Cardiac surgery	401	26 (10,45) mon	53.4	VIS‐operatively and VIS 2 h, VIS 24 h, VIS 48 h postoperatively
Lex et al., 2016 [51]	Australia	Secondary analysis of a single-central prospective	2004–2008	Cardiac surgery	1520	NA	39.1	VIS 72 h after surgery
Algaze et al., 2017 [52]	USA	Retrospective Single-center	2004–2012	Extracardiac Fontan operation with or without CPB	138	3.4 (3.15–4.4) yr	29.7	VIS 0 days after surgery
Siehr et al., 2016 [53]	USA	Retrospective Single-center	2009–2012	Stage 1 surgical palliation consisting of a modified Norwood procedure with right ventricle to pulmonary artery conduit	32	4.6 5± 2.75 days	56.3	VIS 72 h after cardiac surgery
Asfari et al., 2021 [54]	USA	Retrospective Single-center	2012–2018	Cardiac surgery with CPB	121	9 (6–28) days	NA	VIS 0 h, VIS 12 h, VIS 24 h, VIS 36 h, VIS 48 h, VIS 60 h after surgery
Yokota et al., 2022 [55]	USA	Retrospective Single-center	2014–2020	Cardiothoracic surgery with Williams syndrome	70	1.75 (0.7–3.4) yr	37.1	VIS 1 h, VIS 6 h, VIS 12 h, VIS 24 h after surgery
Zhang et al., 2020 [56]	China	Retrospective Single-center	2010–2018	Isolated systemic pulmonary shunt	451	NA	48.1	VIS 24 h after surgery
Radbill et al., 2022 [57]	USA	Prospective Single-central	2007–2019	CHD surgery	1250	8.2±5.8 yr	48.7	max VIS 48 h after surgery
**Adults**								
Yamazaki et al., 2018 [11]	Japan	Retrospective Single-center	2009–2012	Cardiac surgery with CPB	129	61.7 ±17.5 yr	50	At the end of cardiac surgery
Koponen et al., 2019 [10]	Finland	Retrospective Single-center	2010–2014	Cardiac surgery	3213	68 (19–90) yr	27	The first 24 h after ICU arrival
Han et al., 2021 [58]	China	Retrospective Single-center	2015–2021	Cardiac surgery	90	58.0 ±10.7yr	37.8	VIS on the first postoperative day and at the start of RRT
Kwon et al., 2022 [59]	Korea	Retrospective Single-center	2010–2016	Off-pump coronary artery bypass grafting	2149	64.0 (57.0–71.0) yr	22.2	max VIS 48 after surgery
Caballero et al., 2015 [60]	Spain	Retrospective, Multi-center	2000–2009	Emergency heart transplant	390	50 ± 12 yr	19	VIS preoperative
Baysal et al., 2021 [61]	Turkey	Prospective Single-central	2018–2019	Elective on-pump coronary artery bypass grafting	290	62.5 (37–86) yr	23	VIS at the end of the operation
Jiang et al., 2022 [62]	China	Retrospective Single-central	2013–2021	CRRT-therapy patients with cardiac surgery	84	61.0 ± 13.3yr	32.1	VIS 24 h after surgery, VIS 2 h before and after CRRT
Knight et al., 2022 [63]	USA	Retrospective Single-central	2013–2017	BOLTx	245	53 ± 14 yr	45.7	max VIS intraoperative
Liu et al., 2018 [64]	China	Retrospective Single-central	2013–2014	Cardiac surgery	112	59.4 ±11.5 yr	35.7	VIS before commencing NIV
Carmona et al., 2020 [65]	France	Retrospective Single-central	2010–2018	LVAD implantation	68	59.6 ± 13.4 yr	14.7	VIS 24 h after surgery
Poterucha et al., 2019 [66]	USA	Retrospective Single-central	2002–2013	Cardiac surgery	247	33 (18–83) yr	58.7	max VIS 24 h, VIS 48 h after surgery
Sunavsky et al., 2018 [67]	Germany	Retrospective Single-central	2005–2016	Urgent listing for a heart transplant†	434	50 (40.58) yr	21.4	VIS at the time of high urgent registration
Lim et al., 2017 [68]	Korea	Retrospective Single-central	2007–2015	Cardiac surgery	160	66.6 ± 8.6 yr	43.8	VIS 24 h following surgery
Hou et al., 2021 [69]	China	Retrospective Single-central	2017–2019	Cardiovascular surgery	1935	62 (54–69) yr	44.9	max VIS 24 h after surgery
Singh et al., 2022 [70]	India	Retrospective Single-central	2015–2020	OPCABG	687	64.2 ± 9.1 yr	18.1	VIS (mean) intra-, and postoperative
Han et al., 2019 [71]	USA	Retrospective Single-central	2004–2015	LVAD implantation	418	58 ±13 yr	17.9	VIS intra-, and postoperative

Vasoactive-inotropic score (VIS) = dopamine dose (mcg/kg/min) + dobutamine dose (mcg/kg/min) + 100 x epinephrine dose (mcg/kg/min)+ 10 x milrinone dose (mcg/kg/min) + 10,000 x vasopressin dose (units/kg/min) +100 x norepinephrine dose (mcg/kg/min)

*CPB* cardiopulmonary bypass, *ASO* arterial switch operation, *TGA* transposition of the great arteries, *TCPC* total cavopulmonary surgery, *ALCAP* anomalous origin of the left coronary artery from the pulmonary artery surgery, *VA-ECMO* venoarterial extracorporeal membrane oxygenation, *CHD* congenital heart defects, *CRRT* continuous renal replacement therapy, *BOLTx* bilateral orthotopic lung transplant, *CHD* congenital heart disease

**Table2:** Predictive validity of the VIS

Author, year	Outcome variables	ROC area	Sensitivity	Specificity	VIS (high)* cutoff
Tadros et al., 2020 [25]	Primary graft dysfunction	>0.8	0.81	0.74	Max VIS 0–24 h ≥ 10
Garcia et al., 2016 [9]	Composite adverse outcomes	0.762	0.67	0.74	Max VIS 24–48 h > 4.75
Kwon et al., 2022 [59]	1-year mortality	0.82	N/A	N/A	Max VIS 0–48 h > 10.5,
Kim et al., 2015 [26]	Early mortality	0.852	83.3%	85.3%	Preoperative VIS >12.5
Barge-Caballero et al., 2015 [60]	Postoperative infection	N/A	N/A	N/A	VIS at time of transplantation ≥ 20
Baysal et al., 2021 [61]	Early postoperative morbidity and mortality	0.969	0.9	0.88	VIS at the end of the surgery ≥ 5.5
Bangalore et al., 2017 [28]	Length of ICU stay and hospital stay	0.85–0.88	71.4–85.7	75.0–81.9	Mean VIS 0–24 h ≥ 4.5 Max VIS 0–24 h ≥ 4.8 Mean VIS 24–48 h ≥ 3.1
Friedland-Little et al., 2014 [29]	The need for ECMO post-Norwood	0.83	73.9	82.3	Postoperative VIS 0–48 h ≥ 27
Poterucha et al,. 2019 [66]	Early mortality	0.916	72.6	84.3	maxVIS ≥ 3
Gaies et al., 2014 [41]	Composite outcome (death, MCS, RRT, cardiac arrest or CNS injury)	0.79	57	82	Highest VIS 0–24 h ≥ 20
Kumar et al., 2014 [42]	Sepsis; hematological complications; hepatic dysfunction	N/A	N/A	N/A	Highest VIS 0–48 h > 10
Sun et al., 2022 [50]	Prolonged mechanical ventilation	0.780	67.7	83.4	Mean VIS 24–48 ≥ 5.5
Zhang et al., 2020 [56]	Shunt failure after systemic pulmonary shunt	0.84	73	84	VIS 0–24 h ≥ 8.5
Davidson et al., 2012 [8]	Prolonged mechanical ventilation	0.93	N/A	N/A	VIS at 48 h ≥ 10.5; highest VIS 0–48 h ≥ 17; IS at 48h ≥ 3.9; highest IS 0–48 h ≥ 8
Koponen et al., 2019 [10]	Composite outcome (30-day mortality, mediastinitis, cerebral infarction, cerebral hemorrhage, RRT, and myocardial infarction)	N/A	N/A	N/A	Highest VIS 0–24 h > 30
Lim et al., 2017 [68]	NOMI	N/A	53	88	Total VIS 0–24 h ≥ 400
Liu et al., 2018 [64]	NIV failure	N/A	N/A	N/A	VIS before commencing NIV ≥ 6

*Abbreviations*: *ECMO* extracorporeal membrane oxygenation, *MCS* mechanical circulatory support, *RRT* renal replacement therapy, *CNS* central neurologic injury (stroke or seizure), *NOMI* nonocclusive mesenteric ischemia, *NIV* noninvasive ventilation, *VIS* vasoactive-inotropic score, *AUC* area under the receiver operative characteristic (ROC) curve

###  Primary outcomes

**Fig. 2:**
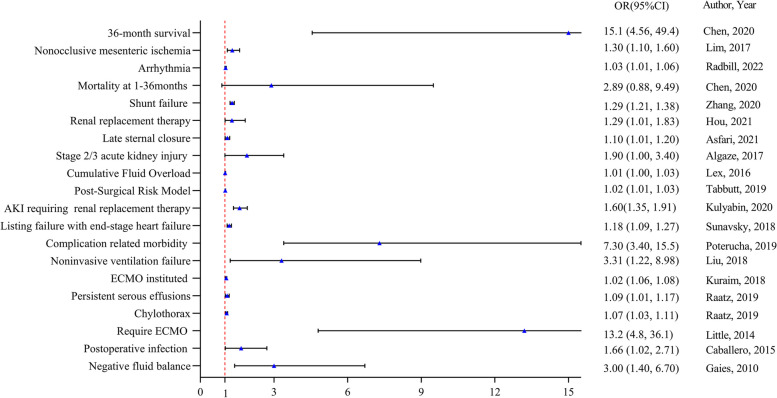
Correlation of the included studies between VIS and outcomes

**Fig. 3:**
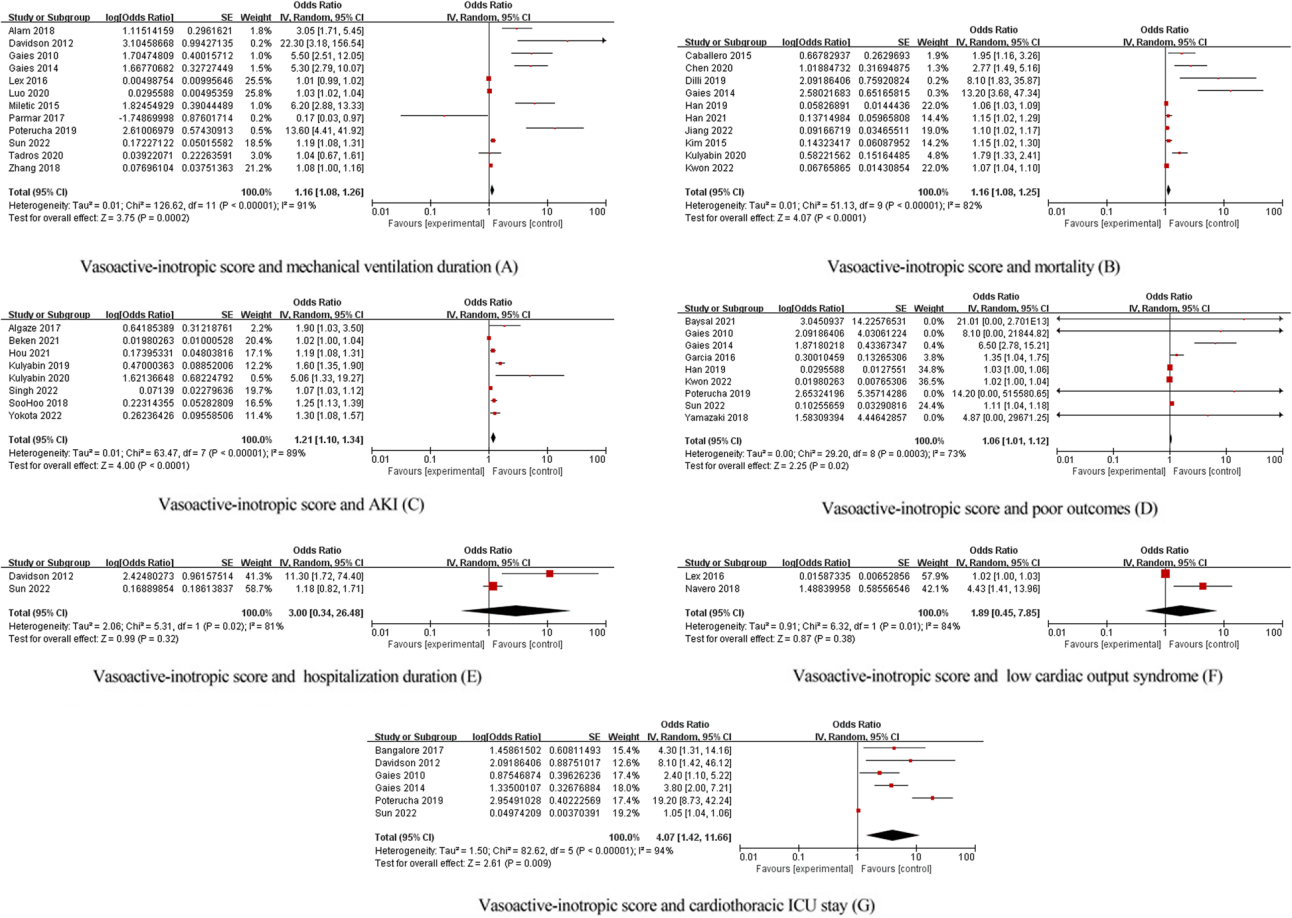
Meta-analysis on association between the VIS and patient outcomes

Studies demonstrating a correlation between the VIS and outcomes such as arrhythmias, nonocclusive mesenteric ischemia (NOMI), extracorporeal membrane oxygenation (ECMO) instituted, chylothorax, or postoperative infection are not pooled estimated odd ratio for only one study provided the effect measures of outcomes concerning the VIS, and we summarized the results of each article individually in Fig. 2. A meta-analysis was conducted if two or more studies provided the same effect measures of outcomes concerning the VIS. The association of VIS with mechanical ventilation duration [18, 25, 27, 34, 35, 37, 41, 44, 50, 51, 66], mortality [3, 26, 36, 41, 43, 58–60, 62, 71], acute kidney injury (AKI) [22, 33, 43, 45, 52, 55, 69, 70], a low cardiac output syndrome (LCOS) [11, 51], LOS in the ICU [1, 8, 28, 41, 50, 66], poor outcomes [1, 9, 11, 41, 50, 59, 61, 66, 71], and LOS in the hospital [8, 50] were meta-analyzed (Fig. 3). We only provided a first step in validating the association between the VIS and patient outcomes and did not consider timepoints. Despite of the timepoints of the VIS being recorded at 24 h, 48 h, and 72 h after surgery, we referred to them collectively as the early postoperative period. It was concluded that the early postoperative VIS is associated with mechanical ventilation duration (OR, 1.16; 95% CI, 1.08, 1.26; *p* < 0.00001), mortality (OR, 1.16; 95% CI, 1.08, 1.25; *p* < 0.00001), AKI (OR, 1.21; 95% CI, 1.10, 1.34; *p* < 0.00001), LOS in the ICU (OR, 4.07; 95% CI, 1.42, 11.66; *p* = 0.009), and poor outcomes (OR, 1.06; 95% CI, 1.01, 1.12; *p* = 0.02), respectively, in Fig. 3A, B, C, G, and D.

###  Sensitivity analysis

**Fig. 4:**
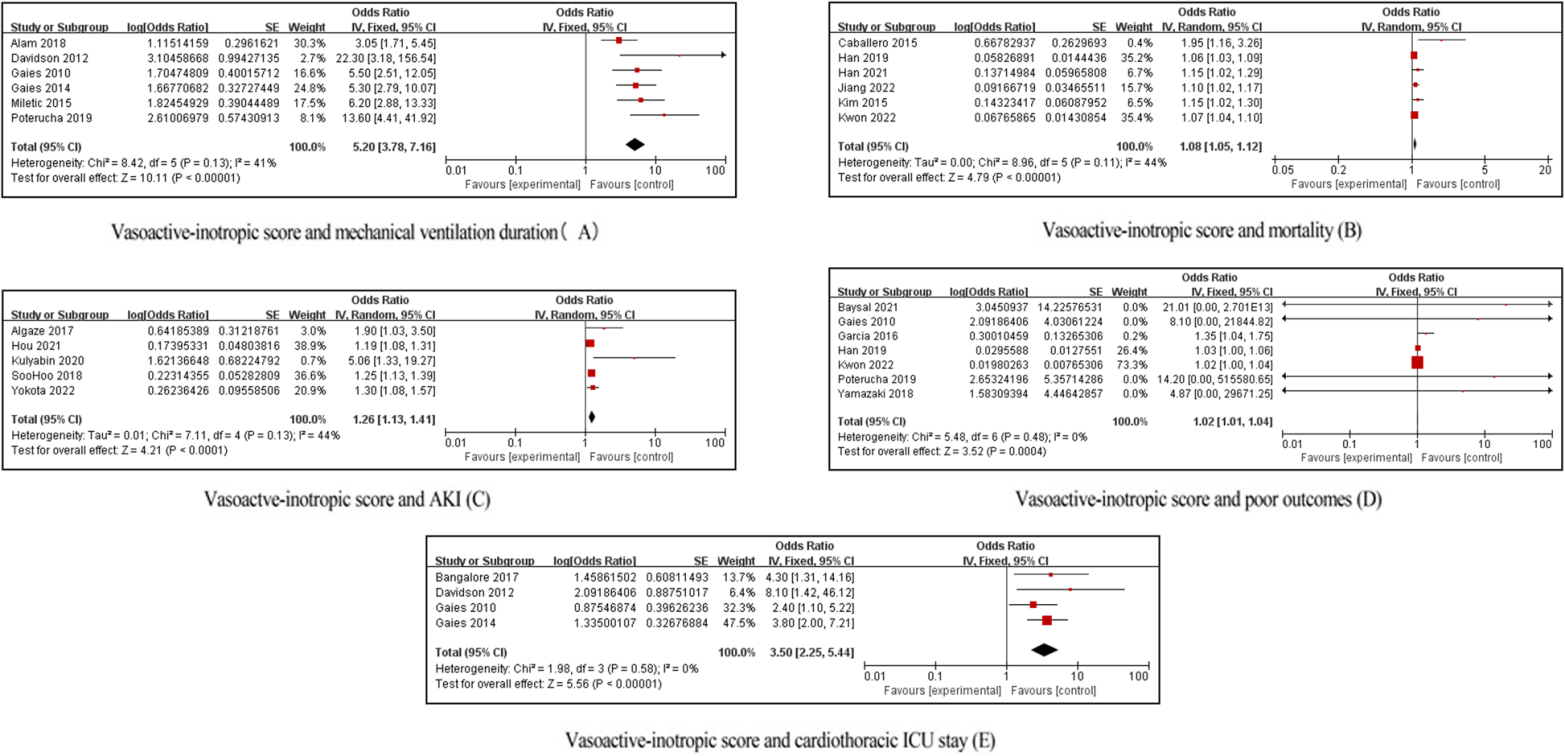
Meta-analysis on association between the VIS and patient outcomes after sensitivity analysis

Considering that significant heterogeneity among studies were detected for mechanical ventilation duration (*I*^2^ = 91%), mortality (*I*^2^ = 82%), AKI ( *I*^2^ = 89%), LOS in the ICU (*I*^2^ = 94%), and poor outcomes (*I*^2^ = 73%), sensitivity analysis using leave one out was conducted. Finally, when omitting the studies of Tadros et al. [25], Lex et al. [51], Luo et al. [34], Parmar et al. [27], Sun et al. [50], and Zhang et al. [35], heterogeneity greatly decreased for mechanical ventilation duration (*I*^2^ = 41%), and significant differences still existed (OR 5.20; 95% CI 3.78, 7.16; *p* < 0.00001) (Fig. 4A). When omitting the studies of Chen et al. [36], Dilli et al. [3], Gaies et al. [41], and Kulyabin et al. [43], heterogeneity greatly decreased for mortality (*I*^2^ = 44%), and significant differences still existed (OR 1.08; 95% CI 1.05, 1.12; *p* < 0.000001) (Fig. 4B). When omitting the studies of Beken et al. [33], Kulyabin et al. [43, 22], and Singh et al. [70], heterogeneity greatly decreased for AKI (*I*^2^ = 44%), and significant differences still existed (OR 1.26; 95% CI 1.13, 1.41; *p* < 0.0001) (Fig. 4C). When omitting the studies of Gaies et al. [41] and Sun et al. [50], heterogeneity disappeared for poor outcomes (*I*^2^ = 0%), and significant differences still existed (OR 1.02; 95% CI 1.01, 1.04; *p* = 0.0004) (Fig. 4D). When omitting the studies of Poterucha et al. [66] and Sun et al. [50], heterogeneity disappeared for LOS in the ICU (*I*^2^ = 0%), and significant differences still existed (OR 3.50; 95% CI 2.25, 5.44; *p* < 0.00001) (Fig. 4E). Considering only two studies were included, LCOS and LOS in the hospital could not undergo sensitivity analysis. The sensitivity analyses, using a leave-one-out approach, suggest that the findings are robust. This is important because the initial *I*^2^ statistics showed high heterogeneity, meaning that the studies were quite different in some way. Removing certain studies reduced this heterogeneity while preserving the statistical significance.

###  Subgroup analysis

**Fig. 5:**
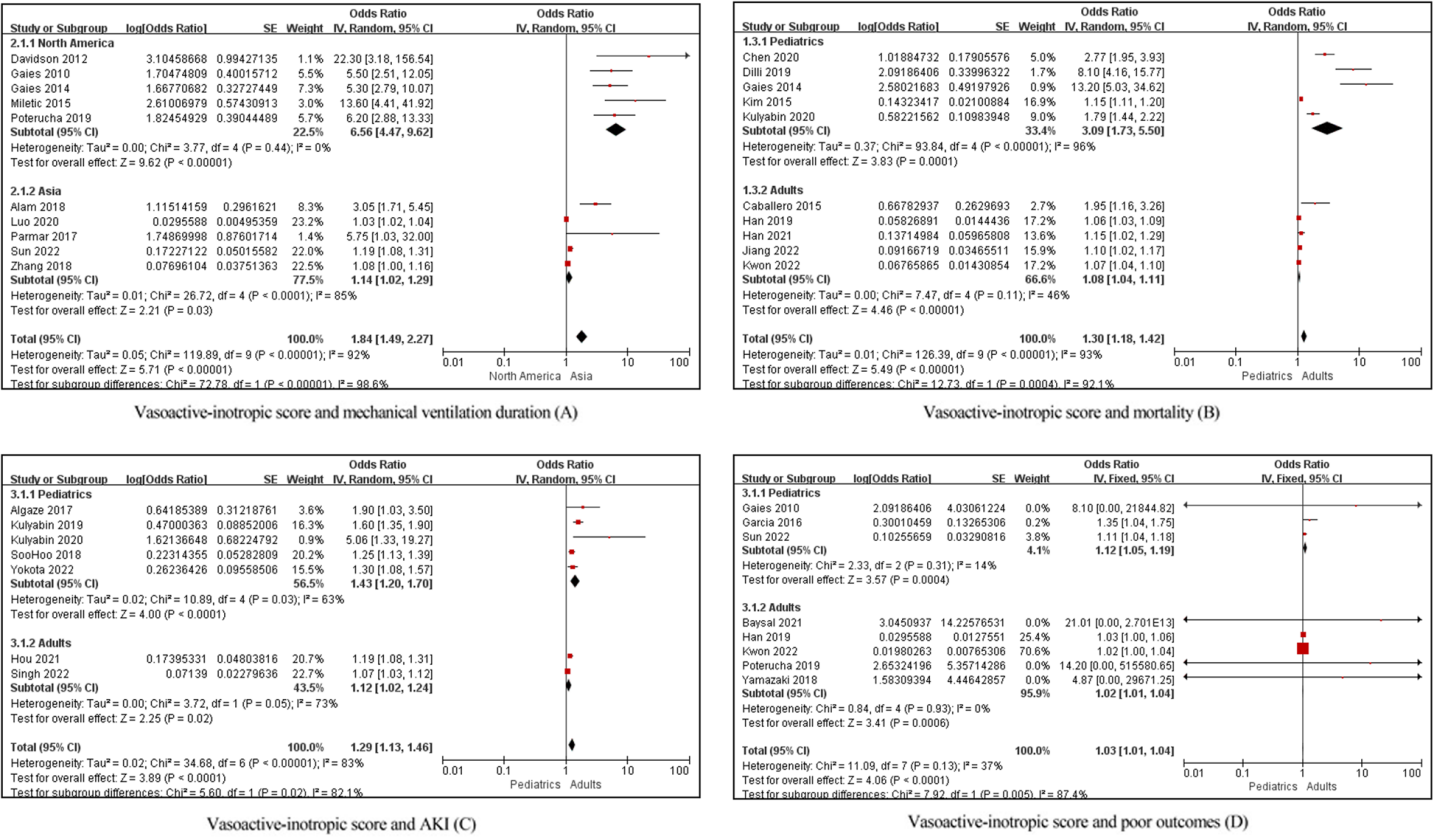
Meta-analysis on association between the VIS and patient outcomes after subgroup analysis

Due to the considerable difference in surgical procedures, timepoints of the VIS, and only one [66] involved adult patients, the association between the VIS and mechanical ventilation duration was only assessed in different continents subgroups. The pooled estimate was different for those in North American (OR 6.56; 95% CI 4.47, 9.62; *p* < 0.000001) compared with those in Asia (OR 1.14; 95% CI 1.02, 1.29; *p* = 0.03) as shown in Fig. 5A. considering difference in study design, timepoints of the VIS, and definition of outcome variables observed, the association of the VIS with mortality, AKI, and poor outcomes was assessed in different age subgroups. For mortality, the pooled estimate was different for pediatrics (OR 3.09; 95% CI 1.73, 5.50; *p* = 0.0001) compared with adults (OR 1.08; 95% CI 1.04, 1.11; *p* < 0.00001) as shown in Fig. 5B. For AKI, the pooled estimate was different for pediatrics (OR 1.43; 95% CI 1.20, 1.70; *p* < 0.0001) compared with adults (OR 1.12; 95% CI 1.02, 1.24; *p* = 0.02) as shown in Fig. 5C. For poor outcomes, the pooled estimate was different for pediatrics (OR 1.12; 95% CI 1.05, 1.19; *p* = 0.0004) compared with adults (OR 1.02; 95% CI 1.01, 1.04; *p* = 0.0006) as shown in Fig. 5D. After age stratification, the heterogeneity decreased. The association of the VIS with mortality was more significant in pediatrics, and age did not affect the association of the VIS with AKI and poor outcomes. There are differences in the strength of the VIS association when looked at from the perspective of geographical location and age groups. This could be indicative of other factors (healthcare system quality, population health characteristics, etc.) affecting the outcomes, and it suggests that the VIS might be more or less useful in different settings or for different age groups.

##  Discussion

In this study, the authors concluded that the elevated VIS in the early postoperative period could predict outcomes, including AKI, mechanical ventilation duration, mortality, poor outcomes, and LOS in the ICU. It is important to note that more than three-quarters of the adverse outcomes developed 24-h postoperative. Therefore, awareness of the VIS from the time of return to the ICU can aid the medical team in risk stratification, targeted interventions, and parental counseling. The VIS, with readily available parameters, is easy to calculate at bedside. As a straightforward hemodynamic observation parameter does not require laboratory examination, it becomes an advantage for medical institutions with limited facilities or in places with grassroots levels to early identify and stratify patients in high-risk cardiovascular surgery. However, careful interpretation is required due to the considerable difference in study design, timepoints of the VIS, and definition of outcome variables observed. In surgical center, patients were typically started on vasoactive‐inotropic agents by the attending anesthesiologist and the cardiac surgeon without uniform protocol. Progress may be affected by the clinical experience of the anesthesiologist and PICU physician. We did not consider the concurrent validity assessed the performance of the VIS in comparison to other scales (such as vasoactive-ventilation-renal (VVR) score [24], total inotrope exposure score(TIES) [28]) which may prove to be a better predictors of patient outcomes in the surgical theater. Despite the low to moderate risk of bias according to JBI checklist scores, there was only one randomized controlled trial included. This could limit the strength of the conclusions. Despite these challenges and limitations, VIS can be used as predictor of patient outcomes in the surgical theater as it is easy and simple to measure without the need for prolonged follow‐up. Our preliminary effort was to merely describe the strength of the association between the VIS and patients outcomes. Understanding how these statistically significant findings translate into clinical practice would be the next step. Prospective randomized trials are necessary to directly correlate the VIS to predicting morbidity and mortality after surgical operations.

The VIS was developed to quantify vasoactive and inotropic support after cardiac surgery in pediatric patients. Gaies et al. [1] analyzed the association between the VIS and clinical outcomes in infants undergoing cardiac surgery and demonstrated that maximum (max) VIS values within the first 48 h postoperatively were associated with poor outcomes in terms of cardiac arrest, circulatory support, renal replacement therapy, neurologic injury, and death. Max VIS values at 24 and 48 h were significantly associated with increased hospitalization and prolonged weaning periods among adolescents (10–18 years) with congenital heart disease [9]. Davidson et al. [8] concluded that higher VIS values at 48 h was strongly associated with increased intubation time and long-term ICU and hospital stay in infants (90 days) after cardiovascular surgery. In comparison with the pediatric population, many more studies were published in adults as well. Kwon et al. [59] found that the increased VIS during the immediate postoperative 48 h following of-pump coronary artery bypass grafting (OPCAB) was significantly associated with long-term morbidity and mortality up to 1 year. A study [60] applied the VIS score in the adult population and demonstrated that the adverse prognostic impact of a high preoperative VIS was especially marked among candidates bridged to transplantation under mechanical circulatory support (MCS). Alam et al. [44] found a higher inotropic requirement (depicted by higher VIS) to be significantly associated with delayed extubation. Hou et al. [69] retrospectively reviewed 1935 adult patients who underwent cardiovascular surgery and showed that the max VIS was associated with postoperative AKI and the need for RRT in AKI patients.

In some studies, no significant association was found between the VIS and outcomes after adjusting for confounders. Knight et al. [63] showed no significant association between the VIS and AKI at 7 days after controlling for covariates in bilateral orthotopic lung transplant recipients. Ödek et al. [39] concluded that lower VIS (*p*<0.05) was associated with early extubation (EE), but when combined into a multivariate model, there was no significantly associated with EE. Talwar [48] analyzed the VIS and found it was not related to mortality in patients who underwent the bidirectional Glenn procedure. Siehr et al. [53] revealed that the VIS was not identified as a significant predictor of hospital length of stay in infants who underwent stage 1 surgical palliation consisting of a modified Norwood procedure with right ventricle to pulmonary artery conduit. Asfari et al. [54] identified that a higher VIS at 36 h was not independently associated with mortality and hospital length of stay. Although no significantly associated was shown in those studies, the results may have been affected by domestic insurance regulations and other issues during the treatment course.

The optimal cutoff value of the VIS as a predictor of adverse outcomes varied from 10 to 30, and none of the studies reported the same VIS cutoff value. This discrepancy in cutoff value is likely due to the differences in the definition of poor outcomes, baseline characteristics, and surgical procedures between studies. Liu et al. [64] found that the VIS ≥ 6 was a risk factor for noninvasive ventilation (NIV) failure in patients with post-extubation ARF after cardiac surgery. In a study by Zhang et al. [56], it was reported that the max VIS over the first 24 h was a good predictor of poor clinical outcome. VIS ≥ 8.5 (OR 1.294, 95% CI 1.210−1.384) was identified as risk factor for shunt failure. Baysal et al. [61] attributed prognostic implications to a postoperative VIS > 5.5 as an independent predictor of morbidity and mortality after coronary artery bypass grafting in their prospective evaluation. At the same time, with the understanding that VIS only allows for the hemodynamic support quantification at a single time-point, the conceptualization of a VIS index by Crow et al. [30], a VIS index ≥ 3 has been outlined to be associated with an increased risk of poor composite outcomes after cardiac surgery.

The VIS seems to have some prognostic value in predicting the potential need for ECMO in the early postoperative period. Xie et al. [72] focused on the VIS at the initiation of ECMO and at the weaning of ECMO to assess recovery of heart function and found that early initiation of ECMO could potentially lead to improved outcomes in these critically ill patients, before inotropic and vasopressor therapy escalation. Kuraim et al. [32], after infant cardiac surgery among 20/565 (3.5%) patients who were placed on veno-arterial ECMO in this early post-operative period after ICU admission, concluded that the highest VIS postoperative day 1 was associated with early ECMO. Friedland-Little et al. [29] identified a peak VIS of 27 within 48 h of surgery as most prognostic of the need for ECMO. The VIS is a good predictor, but further study is needed to determine whether replacing the addition of a third or fourth vasoactive agent with mechanical circulatory assist devices would be more beneficial to the patient’s prognosis. Our findings would serve as hypothesis-generating information to design and conduct prospective trials for validation.

Several possible underlying mechanisms linking the VIS to patients outcomes have been explored. The VIS is used to quantify the amount of cardiovascular support, and a higher VIS score may indicate more severe hemodynamic disorder [1, 41]. Hemodynamic perturbations, especially hypoperfusion, are considered to be important determinant in the development of poor outcomes after cardiovascular surgery [73]. High doses of vasoactive and inotropic medications, particularly catecholamines, have been shown to have a harmful effects on organ function and cause immune-mediated injury [74]. Cardiovascular surgery is highly traumatic, and the postoperative period is prone to excessive bleeding, LCOS, and hypovolemia. These conditions directly manifest in low blood pressure values, leading to reduced organ blood perfusion, which preferentially supplies vital organs such as the heart and brain, causing ischemic perfusion injury and multiple organ failure [58]. Vasoactive medications can increase myocardial oxygen consumption, cardiac arrhythmia, hypertension/hypotension, and peripheral and cardiac ischemia, which may be fatal [75]. Furthermore, catecholamine use has been associated with immunosuppression, bacterial growth, increased bacterial virulence, biofilm formation, insulin resistance, and hyperglycemia [76]. Catecholamine-induced metabolic changes include increased oxygen consumption, glycolysis, glycogenolysis, lipolysis, gluconeogenesis, ketogenesis, peripheral insulin resistance, and lactate release, which can lead to acidosis that may decrease the effect of inotropic or vasopressor drugs and often be a reason for increasing doses [77]. All of these factors can contribute to multiple organ dysfunction, making recovery from cardiovascular surgery more difficult.

In the era of electronic data management systems, it is suggested to program the hourly automatic calculation of the VIS into an electronic medical record system. This would allow for the incorporation start and stop dates as well as dose changes in studying the relationship between multiple clinical parameters and prognosis status over a defined time period in postoperative cardiovascular patients. Meanwhile, with the increasing reliance on digital systems and electronic health records, the potential risks associated with data breaches, unauthorized access, and cyberattacks cannot be overlooked [78, 79].

To properly interpret our study results, it is necessary to understand several limitations: (1) The VIS is a sum of the total vasopressor dose at a single timepoint, without providing information on the duration of exposure and incorporating dose magnitude into the equation. Incorporating duration and magnitude of vasopressor requirements during the postcardiac surgical period could improve the VIS sensitivity for predicting outcomes. (2) In this review and meta-analysis, almost all of the studies included were retrospective, which has certain limitations. In the case control studies, the lack of strategies to address incomplete follow up utilized accounted for main part. In cohort studies, the lack of the exposure period of interest long enough to be meaningful accounted for the main part. In cross-sectional studies, the lack of strategies to deal with confounding factors stated accounted for main part. (3) We only provided a first step in validating the strength of the association between the VIS and patients outcomes and did not consider timepoints. Despite of the timepoint of the VIS being recorded at 24 h, 48 h, and 72 h after surgery, we referred to them collectively as the early postoperative period. This could be a limitation, as the timing of measurements can sometimes affect the outcome. (4) We did not consider the concurrent validity assessed the performance of the VIS in comparison to other scales (such as SAPS III, OHCA score, RACA score, EuroSCORE, MR-proADM, APACHE II, SNAPPE-II, Aristotle basic complexity score (ABC), IS, APACHE III score, SOFA score) which also could be used for predicting outcomes in cardiovascular patients. (5) In surgical center, patients were typically started on vasoactive-inotropic agents by the attending anesthesiologist and the cardiac surgeon without uniform protocol. Progress may be affected by the clinical experience of the anesthesiologist and PICU physician. (6) Apart from two multi-center studies [45, 60], all other included studies were single center, and we only included studies published in English, thus affecting the methodological quality of this review and potential publication bias. The applicability of this meta-analysis to the broader patient population may be limited given that most studies involved were conducted in specific countries. At the same time, incomplete retrieval of identified research affected the reporting bias.

##  Conclusions

A higher VIS in the early postoperative period could predict outcomes, the VIS, which is easily calculated from routine work, could assist in predicting outcomes in high-risk cardiovascular surgery and may aid in risk stratification and targeted interventions. However, further prospective studies are required to assess its prognostic value, to validate its association with major adverse events, and to specify optimal doses and combinations of vasoactive inotropic medications.

### Supplementary Information

**Additional file 1:** **Table S1. **Summary of quality assessment for cross-sectional studies (*n*=20). **Table S2.** Summary of quality assessment for cohort studies (*n*=31). **Table S3.** Summary of quality assessment for case control studies (*n*=5).

## Data Availability

The data that support the findings of this study are available on request from the corresponding author.
