# Supplementary material for: The association of vasoactive-inotropic score and surgical patients’ outcomes: a systematic review and meta-analysis
Source: Syst Rev. 2024 Jan 6;13:20. doi: 10.1186/s13643-023-02403-1 (PMC10770946; doi:10.1186/s13643-023-02403-1)
Supplement: Supplementary file 1 — Additional file 1: Table S1. Summary of quality assessment for cross-sectional studies (n=20). Table S2. Summary of quality assessment for cohort studies (n=31). Table S3. Summary of quality assessment for case control studies (n=5). [file 13643_2023_2403_MOESM1_ESM.docx]

**Table S1.** Summary of quality assessment for cross-sectional studies (n=20)

| **Author, Year** | **1. Clearly defined inclusion criteria** | **2. Residents and setting described in detail** | **3.**  **Exposure measured in a valid and reliable way** | **4. Objective, standard criteria used for condition measurement** | **5. Confounding factors identified** | **6. Strategies to deal with confounding factors stated** | **7. Outcomes measured in a valid and reliable way** | **8.**  **Statistical analysis appropriate** | **Total score** |
| --- | --- | --- | --- | --- | --- | --- | --- | --- | --- |
| Gaiesl et al, 2010 | Yes | Yes | Yes | Yes | Yes | Yes | Yes | Yes | 8 |
| Navero et al, 2018 | Yes | Yes | Yes | Yes | Yes | Yes | Yes | Yes | 8 |
| Han et al, 2021 | Yes | Yes | Yes | Yes | Yes | No | Yes | Yes | 7 |
| Hanna et al, 2020 | Yes | Yes | Yes | Yes | Yes | No | Yes | Yes | 7 |
| Garcia et al, 2015 | Yes | Yes | Yes | Yes | Yes | Yes | Yes | Yes | 8 |
| Kim et al, 2014 | Yes | Yes | Yes | NA | Yes | NA | Yes | Yes | 6 |
| Caballero et al, 2015 | Unclear | Yes | Yes | Yes | Yes | NA | Yes | Yes | 6 |
| Bangalore et al, 2017 | Unclear | Yes | Yes | Yes | Yes | Yes | Yes | Yes | 7 |
| Crow et al, 2014 | Unclear | Yes | Yes | Yes | Yes | NA | Yes | Yes | 6 |
| Raatz et al, 2019 | Unclear | Yes | Yes | Yes | Yes | Yes | Yes | Yes | 7 |
| Jiang et al, 2022 | Yes | Yes | Yes | Yes | Yes | NA | Yes | Yes | 7 |
| Knight et al, 2022 | Yes | Yes | Yes | Yes | Yes | NA | Yes | Yes | 7 |
| Liu et al, 2018 | Yes | Yes | Yes | Yes | Yes | NA | Yes | Yes | 7 |
| Carmona et al, 2020 | Yes | Yes | Yes | Yes | Yes | NA | Yes | Yes | 7 |
| Joseph et al, 2018 | Unclear | Yes | Yes | Yes | Yes | Yes | Yes | Yes | 7 |
| Chen et al, 2020 | Unclear | Yes | Yes | Yes | Yes | Yes | Yes | Yes | 7 |
| Tabbutt et al, 2019 | Yes | Yes | Yes | Yes | Yes | Yes | Yes | Yes | 8 |
| Campbell et al, 2020 | Yes | Yes | Yes | Yes | No | No | Yes | Yes | 6 |
| Talwar et al, 2017 | Yes | Yes | Yes | Yes | No | No | Yes | Yes | 6 |
| Han et al, 2019 | Yes | Yes | Yes | Yes | Yes | Yes | Yes | Yes | 8 |

**Table S2.** Summary of quality assessment for cohort studies (n=31)

| **Author, Year** | **1. Similar study groups recruited from same population** | **2. Exposures measured similarly in assignment to groups** | **3. Exposure measured in a valid and reliable way** | **4. Confounding factors identified** | **5. Strategies to deal with confounding factors stated** | **6. Residents free of outcome at start of study** | **7. Outcomes measured in a valid and reliable way** | **8. Follow-up time reported** | **9. Follow-up complete. If not, reasons described** | **10. Strategies to address incomplete follow up utilized** | **11. Statistical analysis appropriate** | **Total score** |
| --- | --- | --- | --- | --- | --- | --- | --- | --- | --- | --- | --- | --- |
| Yamazaki et al, 2016 | Yes | Yes | Yes | Yes | Yes | Yes | Yes | Yes | Yes | NA | Yes | 10 |
| Koponen et al, 2019 | Yes | Yes | Yes | Yes | Yes | Yes | Yes | Yes | Yes | NA | Yes | 10 |
| Dilli et al, 2019 | Yes | Yes | Yes | Yes | Yes | Yes | Yes | Yes | Yes | NA | Yes | 10 |
| Davidson et al, 2012 | Yes | Yes | Yes | Yes | Yes | Yes | Yes | Yes | Yes | NA | Yes | 10 |
| Scherer et al, 2016 | Yes | Yes | Yes | Yes | Yes | Yes | Yes | NA | NA | NA | Yes | 8 |
| Kwon et al, 2022 | Yes | Yes | Yes | Yes | Yes | Yes | Yes | Yes | NA | NA | Yes | 9 |
| Parmar et al, 2017 | Yes | Yes | Unclear | Yes | NA | Yes | Yes | NA | NA | NA | Yes | 6 |
| Baysal et al, 2021 | Yes | Yes | Unclear | Yes | NA | Yes | Unclear | NA | NA | NA | Yes | 5 |
| Kuraim et al, 2018 | Yes | Yes | Yes | Yes | Yes | Yes | Yes | Yes | Yes | Unclear | Yes | 10 |
| Beken et al, 2020 | Yes | Yes | Yes | NA | No | Yes | Yes | Yes | Yes | NA | Yes | 8 |
| Schroeder et al, 2018 | Yes | Yes | Yes | NA | No | Yes | Yes | NA | NA | NA | Yes | 6 |
| Miletic et al,, 2015 | Yes | Yes | Yes | Yes | Yes | Yes | Yes | Yes | NA | NA | Yes | 9 |
| Murin et al, 2018 | Yes | Yes | Yes | NA | No | Yes | Yes | NA | NA | NA | Yes | 6 |
| Ödek et al, 2016 | Yes | Yes | Yes | Yes | Yes | Yes | Yes | Yes | Yes | NA | Yes | 10 |
| Sanil et al, 2013 | Yes | Yes | Yes | Yes | Yes | Yes | Yes | Yes | Yes | NA | Yes | 10 |
| Gaies et al, 2014 | Yes | Yes | Yes | Yes | No | Yes | Yes | Yes | Yes | NA | Yes | 9 |
| Kumar et al, 2014 | Yes | Yes | Yes | Yes | Yes | Yes | Yes | Yes | Unclear | NA | Yes | 9 |
| Kulyabin et al, 2019 | Yes | Yes | Yes | NA | No | Yes | Yes | Yes | Yes | NA | Yes | 8 |
| Alam et al, 2018 | Yes | Yes | Yes | Yes | No | Yes | Yes | Yes | Yes | NA | Yes | 9 |
| SooHoo et al, 2018 | Yes | Yes | Yes | Yes | No | Yes | Yes | Yes | Yes | NA | Yes | 9 |
| Ödek et al, 2018 | Yes | Yes | Yes | Yes | No | Yes | Yes | Yes | Yes | NA | Yes | 9 |
| Sun et al, 2022 | Yes | Yes | Yes | Yes | No | Yes | Yes | Yes | Yes | No | Yes | 9 |
| Lex et al, 2016 | Yes | Yes | Yes | Yes | Yes | Yes | Yes | Yes | Yes | NA | Yes | 10 |
| Algaze et al, 2017 | Yes | Yes | Yes | Yes | Yes | Yes | Yes | Yes | Yes | No | Yes | 10 |
| Siehr et al, 2016 | Yes | Yes | Yes | Yes | No | Yes | Yes | Yes | Yes | NA | Yes | 9 |
| Asfari et al, 2021 | Yes | Yes | Yes | Yes | No | Yes | Yes | Yes | Yes | NA | Yes | 9 |
| Hou et al, 2021 | Yes | Yes | Yes | Yes | Yes | Yes | Yes | Yes | Yes | NA | Yes | 10 |
| Yokota et al, 2022 | No | No | Yes | Yes | No | Yes | Yes | Yes | Yes | NA | Yes | 7 |
| Zhang et al, 2020 | Yes | Yes | Yes | Yes | Yes | Yes | Yes | Yes | Yes | NA | Yes | 10 |
| Singh et al, 2022 | Yes | Yes | Yes | Yes | No | Yes | Yes | Yes | Yes | NA | Yes | 9 |
| Radbill et al, 2022 | Yes | Yes | Yes | Yes | No | Yes | Yes | Yes | Yes | NA | Yes | 9 |

**Table S3.** Summary of quality assessment for case control studies (n=5)

| **Author, Year** | **1**  **Case control groups are comparable** | **2**  **Cases and controls matched appropriately** | **3**  **Same criteria used for identification** | **4**  **Exposure measured in a valid and reliable way** | **5**  **Exposure measured in the same way** | **6**  **Confounding factors identified** | **7**  **Confounding factors identified** | **8**  **Outcomes measured in a valid and reliable way** | **9**  **Was the exposure period of interest long enough to be meaningful** | **10**  **Statistical analysis appropriate** | **Total score** |
| --- | --- | --- | --- | --- | --- | --- | --- | --- | --- | --- | --- |
| Joshua et al, 2013 | Yes | Yes | Yes | Unclear | Unclear | Yes | No | Yes | NA | Yes | 6 |
| Luo et al, 2019 | Yes | Yes | Yes | Yes | Yes | Yes | No | Unclear | NA | Yes | 7 |
| Zhang et al, 2018 | Yes | NA | Yes | Yes | Yes | Yes | No | Yes | NA | Yes | 7 |
| Sunavsky et al, 2018 | Yes | Yes | Yes | Yes | Yes | Yes | No | Yes | Yes | Yes | 9 |
| Lim et al, 2017 | Yes | Yes | Yes | Yes | Yes | Yes | Yes | Yes | Yes | Yes | 10 |
